# Prefrontal Functional Connectivity During the Verbal Fluency Task in Patients With Major Depressive Disorder: A Functional Near-Infrared Spectroscopy Study

**DOI:** 10.3389/fpsyt.2021.659814

**Published:** 2021-05-21

**Authors:** Suh-Yeon Dong, JongKwan Choi, Yeonsoo Park, Seung Yeon Baik, Minjee Jung, Yourim Kim, Seung-Hwan Lee

**Affiliations:** ^1^Department of Information Technology Engineering, Sookmyung Women's University, Seoul, South Korea; ^2^OBELAB, Seoul, South Korea; ^3^Department of Psychology, University of Notre Dame, Dame, RI, United States; ^4^Department of Psychology, Penn State University, State College, PA, United States; ^5^Clinical Emotion and Cognition Research Laboratory, Inje University, Goyang, South Korea; ^6^Department of Psychology, University of Wisconsin-Milwaukee, Milwaukee, WI, United States; ^7^Department of Psychiatry, Inje University Ilsan Paik Hospital, Goyang, South Korea

**Keywords:** major depressive disorder, fNIRS, functional connectivity, verbal fluency task, efficiency

## Abstract

Deviations in activation patterns and functional connectivity have been observed in patients with major depressive disorder (MDD) with prefrontal hemodynamics of patients compared with healthy individuals. The graph-theoretical approach provides useful network metrics for evaluating functional connectivity. The evaluation of functional connectivity during a cognitive task can be used to explain the neurocognitive mechanism underlying the cognitive impairments caused by depression. Overall, 31 patients with MDD and 43 healthy individuals completed a verbal fluency task (VFT) while wearing a head-mounted functional near-infrared spectroscopy (fNIRS) devices. Hemodynamics and functional connectivity across eight prefrontal subregions in the two groups were analyzed and compared. We observed a reduction in prefrontal activation and weaker overall and interhemispheric subregion-wise correlations in the patient group compared with corresponding values in the control group. Moreover, efficiency, the network measure related to the effectiveness of information transfer, showed a significant between-group difference [t (71.64) = 3.66, corrected p < 0.001] along with a strong negative correlation with depression severity (rho = −0.30, *p* = 0.009). The patterns of prefrontal functional connectivity differed significantly between the patient and control groups during the VFT. Network measures can quantitatively characterize the reduction in functional connectivity caused by depression. The efficiency of the functional network may play an important role in the understanding of depressive symptoms.

## Introduction

Impairments in cognitive functions, such as attention, working memory, and executive function, have been reportedly observed in patients with major depressive disorder (MDD). Studies comparing patients and healthy controls have reported that these impairments are associated with abnormal patterns of brain activity. When compared to healthy individuals, patients with MDD have been reported to display aberrant neuropsychological characteristics. Earlier studies have used functional magnetic resonance imaging (fMRI) to reveal reduced prefrontal activation during digit-sorting tasks (1) and verbal fluency tasks (VFTs) (2), imbalance between left and right prefrontal activation during emotional judgment (3), and reduced activation in the right nucleus accumbens during the monetary incentive delay task (4).

The association between prefrontal cortex (PFC) activation and depressive symptoms has also been investigated using near-infrared spectroscopy (NIRS). Multichannel NIRS is an emerging neuroimaging tool that in addition to detecting the spatiotemporal characteristics of brain function using a non-invasive, portable, and restraint-free technique (5), has been validated in psychiatry patients (6). Recently, studies using NIRS have reported a reduction in prefrontal hemodynamic activation in patients with MDD during the VFT, along with a strong negative correlation between the severity of depression and changes in oxygenated hemoglobin (HbO2) (7–9). However, the functional connectivity has not yet been investigated in depth. Some recent studies have characterized distinct resting-state MRI based-functional connectivity in patients with MDD (10), apathetic depression (11), and remitted MDD (12). Kawano et al. reported a strong negative correlation between the HAMD-21 score and average HbO2 concentration in the frontal lobe (9); however, they did not assess the functional connectivity.

Since the human brain consists of a complex network responsible for its function, considerable attention has been given to the graph theory approach for explaining functional deviations in brain organization resulting from neurological disorders such as schizophrenia (13–15), Alzheimer's disease (16, 17), bipolar disorder (18), attention-deficit hyperactivity disorder (19), and depression (20, 21). Although a majority of the earlier studies on functional connectivity relied on fMRI and electroencephalography (EEG) (21), functional NIRS (fNIRS) can be considered as a reasonable alternative for detecting changes in brain functional networks, owing to its high temporal resolution. A recent review paper on the application of fNIRS in MDD research provided comprehensive evidence that the functional connectivity measured by fNIRS is potentially useful for assessing MDD (22). Zhu et al. reported that patients with affective disorders exhibit significantly reduced connectivity in the PFC using resting-state fNIRS (23). Rosenbaum et al. conducted a functional connectivity analysis on patients with late-life depression (24) and further examined functional connectivity in the cortical areas of the default mode network (25). However, to the best of our knowledge, no studies have investigated the differences in functional connectivity during the VFT task.

In this study, we sought to perform an fNIRS-based functional connectivity analysis to investigate PFC activation patterns during the VFT. Unlike the aforementioned studies, which mainly focused on resting-state functional connectivity, we hypothesized that the functional connectivity elicited by cognitive stimulation (using the VFT in this case) differs between patients with MDD and healthy individuals, and that these differences can be visually described by the prefrontal functional network. As a decrease in verbal fluency has been primarily found in patients with MDD, functional connectivity during the VFT may play an important role in characterizing MDD. Moreover, we calculated graph theory-based connectivity metrics to quantitatively characterize functional connectivity in each group. We identified the measures that demonstrated significant between-group differences based on the connectivity metrics.

## Materials and Methods

### Participants

We enrolled 43 healthy participants (age: 34.26 ± 12.47 years, control group) and 31 patients (age: 39.48 ± 13.81 years, patient group) with MDD who were undergoing treatment at the university hospital. The participants in the patient group had already been diagnosed with MDD according to the Diagnostic and Statistical Manual of Mental Disorders, Fifth Edition (26) by a board-certified psychiatrist. The participants' symptoms of depression and anxiety were assessed using the Hamilton Depression Rating Scale (HAMD) (27), Hamilton Anxiety Rating Scale (HAMA) (28), Beck Depression Inventory (BDI-II) (29), and State-Trait Anxiety Inventory (STAI) (30), which was composed of two subscales (STAI-state and STAI-trait, respectively). We excluded patients with an HAMD scores <8 and healthy individuals with an HAMD scores > 8.

Table 1 represent the demographic data for all enrolled participants. All participants were recruited from the Department of Psychiatry, Inje University Ilsan Paik Hospital. The study was conducted in accordance with the Declaration of Helsinki and was approved by the Institutional Review Board of Inje University Ilsan Paik Hospital (2017–10–013). All the participants provided written informed consent prior to undergoing the fNIRS experiment.

**Table 1:** Participant demographic data (*N* = 74).

**Variable**	**Control (*N* = 43) Mean (SD)**	**Patient (*N* = 31) Mean (SD)**	***p*-value**
Age (years)	34.26 (12.47)	39.48 (13.82)	0.093
HAMD	2.02 (1.99)	23.55 (9.47)	<0.001
HAMA	1.42 (1.67)	22.32 (10.78)	<0.001
BDI-II	5.33 (3.96)	25.48 (12.21)	<0.001
STAI-state	31.44 (6.75)	54.94 (11.98)	<0.001
STAI-trait	35.49 (8.78)	59.58 (11.99)	<0.001

*HAMD, hamilton rating scale for depression; HAMA, hamilton rating scale for anxiety; BDI-II, beck depression inventory; STAI, state-trait anxiety inventory*.

### NIRS Device

A head-mounted wireless NIRS system (NIRSIT; OBELAB Inc., Seoul, Korea) was used to obtain HbO2 values from the prefrontal region of the human brain. The NIRS system consists of 24 lasers sources (780/850 nm; maximum power under 1 mW) and 32 photodetectors with multiple source-detector spacing (1.5, 2.12, 3.0, 3.35 cm) resulting in 204 measurement points at a sampling rate of 8.138 Hz (31). We used 48 channels created by a 3.0 cm separation between the source and the detector for further analysis. We aligned the marking point on the front of the device to the center between the eyes to ensure that the same brain region was recorded. Before wearing the device, the subject's hair were finger-combed away from the forehead to minimize its influence on the measurements. After fixing the device position from the front, the strap and the Velcro hooks on the back were securely fixed to prevent the device from moving while recording.

### Activation Task

Participants were instructed to perform the VFT while wearing the wireless head-mounted fNIRS device. The VFT is a type of cognitive assessment, in which participants are asked to produce as many words as possible from a category (semantic or phonemic) in a given period of time. In this study, phonemic VFT was conducted. This task is usually employed in psychological or neuropsychological assessments to detect cognitive impairment (32, 33). The entire procedure consisted of three blocks; each block had a 30-s initial rest and a 60-s VFT (30-s pre-task and 30-s task). Participants were asked to repeat aloud the four Korean consonants, “g,” “n,” “d,” and “r” during the pre-task. During the task, participants were instructed to produce as many Korean words as possible beginning with a designated consonant (randomly presented among the eight consonants, “g,” “n,” “d,” “r,” “m,” “b,” “s,” and “h”).

### Data Analysis

**Figure 1:**
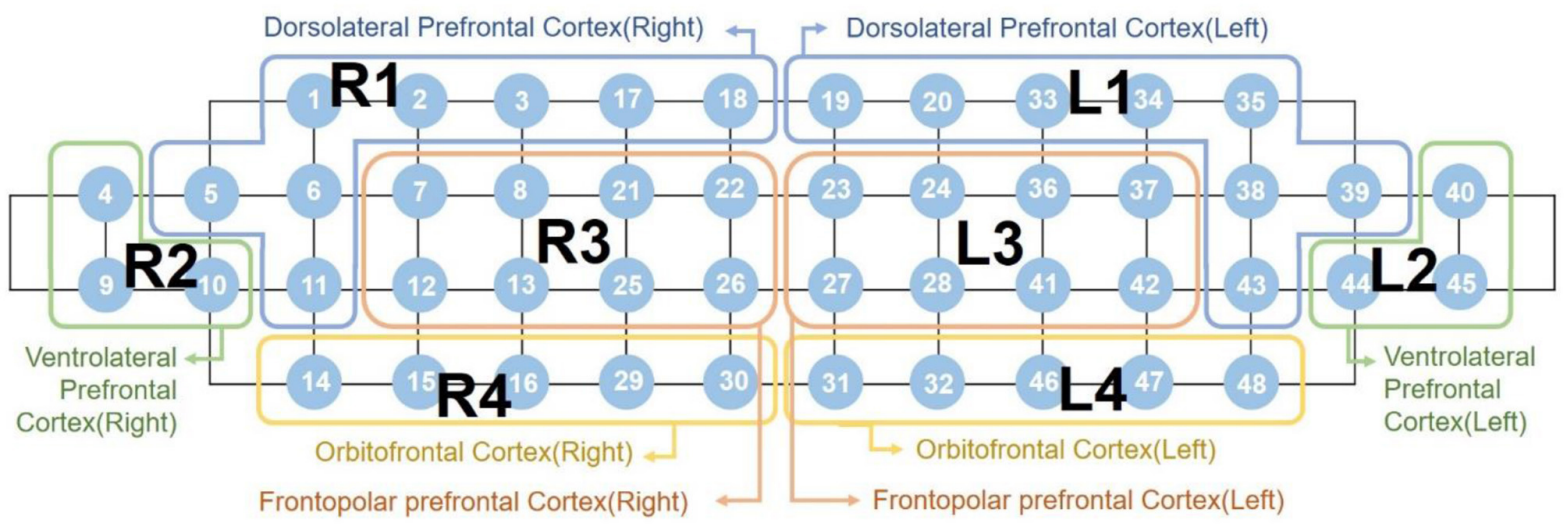
Labeling of the eight subregions mapped by 48 channels “R” indicates the right hemisphere, while “L” indicates the left hemisphere.

The detected light signals were filtered using high and low-pass filters at 0.005 Hz and 0.1 Hz, respectively, to eliminate cardiovascular artifacts and environmental noise. We rejected poor-quality channels with a signal-to-noise ratio under 30 dB prior to extraction of the hemodynamic data to prevent misinterpretation. The location of the 48 NIRS channels with color-scaled rejection ratios of each subject group are provided in Supplementary Figure 1. Subsequently, the hemodynamic responses were extracted using the modified Beer-Lambert law (34) and baseline corrected with the last 5 s of the pre-task as baseline for each block. The average amplitude of the baseline was subtracted. Following baseline correction, we obtained the block averaged responses for each channel. As mentioned in Section 2.2, we only used 48 channels in the prefrontal region. As shown in Figure 1, the 48 channels were grouped into eight subregions: the right and left dorsolateral PFC (labeled R1 and L1); the ventrolateral PFC (R2 and L2); the frontopolar PFC (R3 and L3); and the orbitofrontal cortex (R4 and L4). We obtained averaged hemodynamic responses within each subregion, such that each participant's recording became a N-by-8 matrix wherein N indicated time-domain sample numbers and 8 indicated the number of subregions.

The hemodynamic parameters obtained were analyzed to evaluate the functional connectivity. Specifically, functional connectivity was measured using the strength of temporal correlation of hemodynamics between every possible pair of the subregions. Subsequently, computed prefrontal correlation coefficients were used to extract network measures to represent the global network characteristics, such as density, clustering coefficient, and efficiency. We applied weight thresholding to the correlation coefficients to quantitatively measure and compare the functional connectivity between the two groups. Correlations below the threshold value were set to 0 and were assumed to be weak and non-significant links across a threshold range of 0 to 0.95 with increments of 0.05, as they could have been spurious connections. Suprathreshold correlations were set to 1; therefore, we were able to obtain a binary adjacency matrix for each threshold value (35). Subsequently, we calculated the three most commonly used global connectivity measures as functions of the threshold value, namely, density, clustering coefficient, and efficiency. Data processing and analysis were performed using MATLAB 2018b (Mathworks, Inc., Natick, MA) and network measures were extracted using the brain connectivity toolbox, which is built into the MATLAB environment (36).

### Statistical Analysis

We used a two-sample *t*-test to assess between-group differences in age, psychological scores, representative means, and functional connectivity measures. Homogeneity of variance was assessed using Levene's test for the equality of variance in a two-sample *t*-test. The degree of freedom was adjusted if the equality of variance was not assumed based on the results of Levene's test. Functional connectivity analysis was performed by calculating Pearson's correlation coefficients between every pair of the represented mean values for the eight subregions. *P*-values obtained from the statistical tests were corrected using the false discovery rate (FDR) for multiple comparisons (37). Following the FDR correction, *p*-values under 0.05 were considered statistically significant. All statistical analyses were performed using IBM SPSS Statistics 23 (IBM Corp. Armonk, NY).

## Results

### Age and Psychological Test Scores

First of all, the two groups in this study had no significant differences in age [t (72) = −1.701, p = 0.093]. However, the comparison of the five psychological test scores using two-sample *t*-tests revealed statistically significant between-group differences. The HAMD scores in the patient group (mean ± standard deviation: 23.55 ± 9.47) were significantly higher than those in the control group (2.02 ± 2.00) [t (31.93) = −12.46, corrected *p* < 0.001]. Similarly, the HAMA scores in the patient group (22.32 ± 10.78) were significantly higher than those in the control group (1.42 ± 1.67) [t (31.03) = −10.70, corrected *p* < 0.001]. Moreover, the remaining three scores showed statistically significant between-group differences [BDI-II, t (34.59) = −8.86, corrected *p* < 0.001; STAI-state, t (43.66) = −9.85, corrected *p* < 0.001; STAI-trait, t (72) = −9.99, corrected *p* < 0.001].

### Task Performance

We measured the number of correct words and the reaction time as task performance metrics during each VFT task. Between-group differences were assessed using two-sample *t*-tests. The word counts in the control group (25.40 ± 6.34) were significantly higher than those in the patient group (19.77 ± 7.53) [t (71) = 3.48, *p* = 0.001]. Similarly, reaction times in the control group (3,257.84 ± 711.64 msec) were significantly lower than those in the patient group (4,368.26 ± 1,995.05 msec) [t (35.54) = −2.97, *p* = 0.005].

### fNIRS Activation

**Figure 2:**
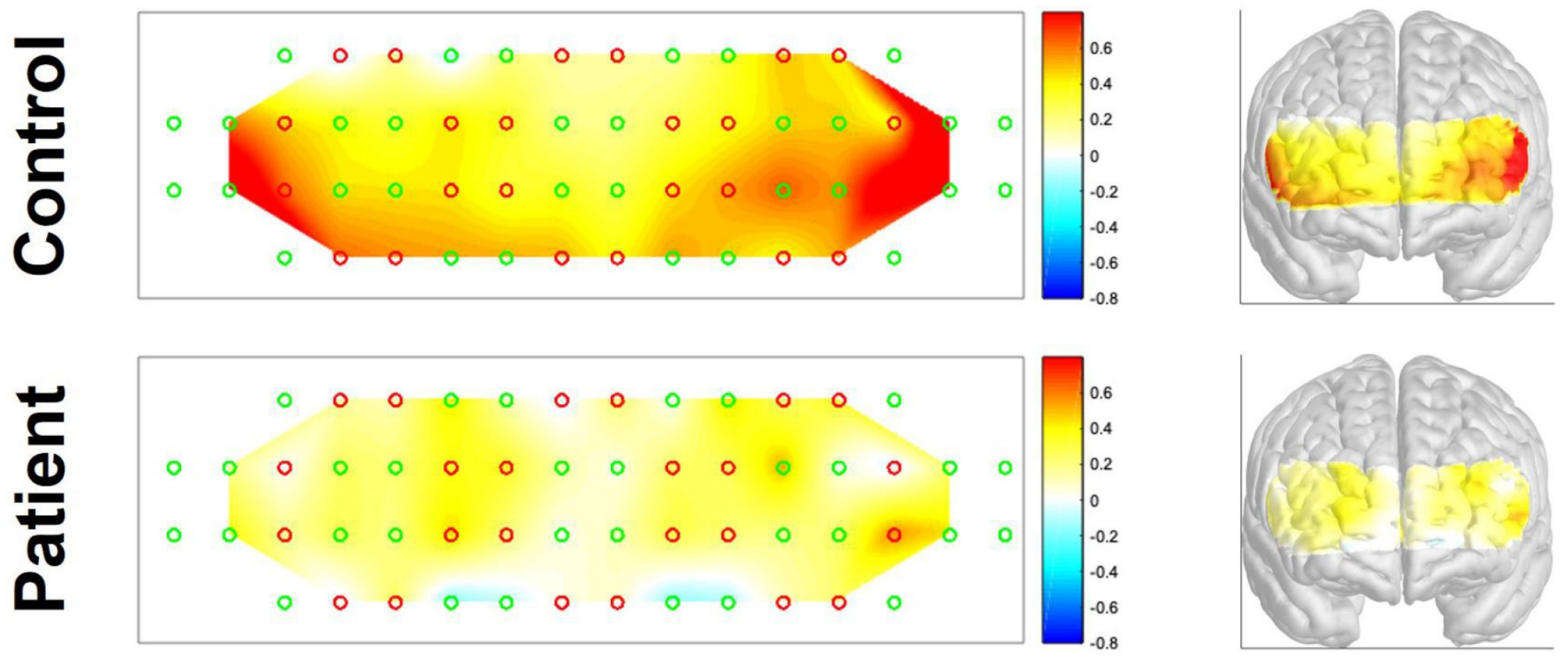
Activation maps of the two groups during the verbal fluency task.

**Figure 3:**
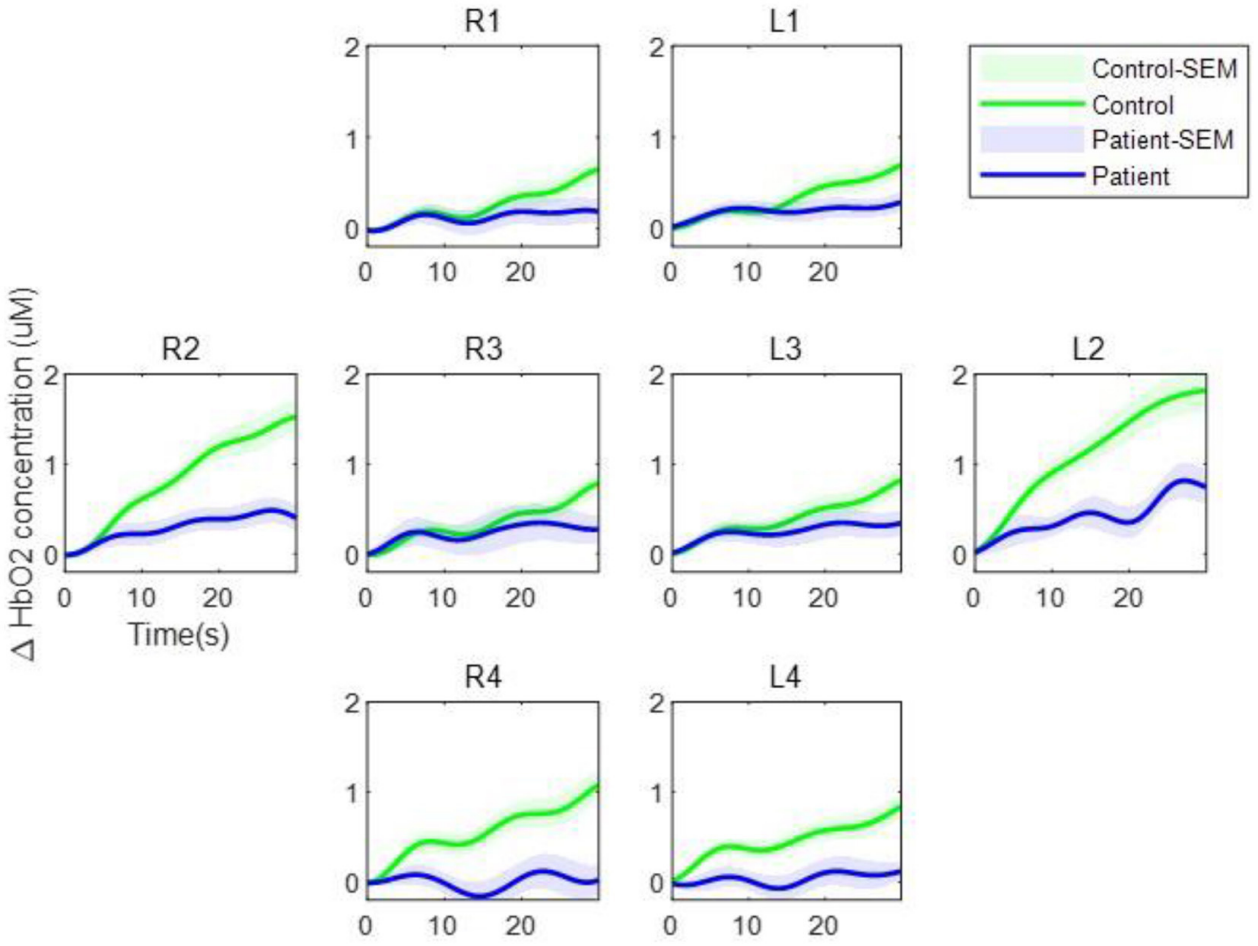
Transient oxygenated hemoglobin concentration during the verbal fluency task. The green line represents the control group while the blue line represents the patient group. The shaded regions represent the standard errors of the mean.

Figure 2 shows the average hemodynamic responses during the VFT in each group. As shown in the figure, the HbO2 concentration during the VFT was higher in the control group than in the patient group. Figure 3 illustrates the transient hemodynamic responses in each subgroup. The transients of the 48 channels are provided in Supplementary Figure 2. During the task period, the control group showed significantly higher HbO2 concentrations than the patient group and returned to baseline after the task (not shown in the figure). Moreover, the gap between the two groups was greater in R2, R4, L2, and L4 compared with the other subregions.

### Functional Connectivity

**Figure 4:**
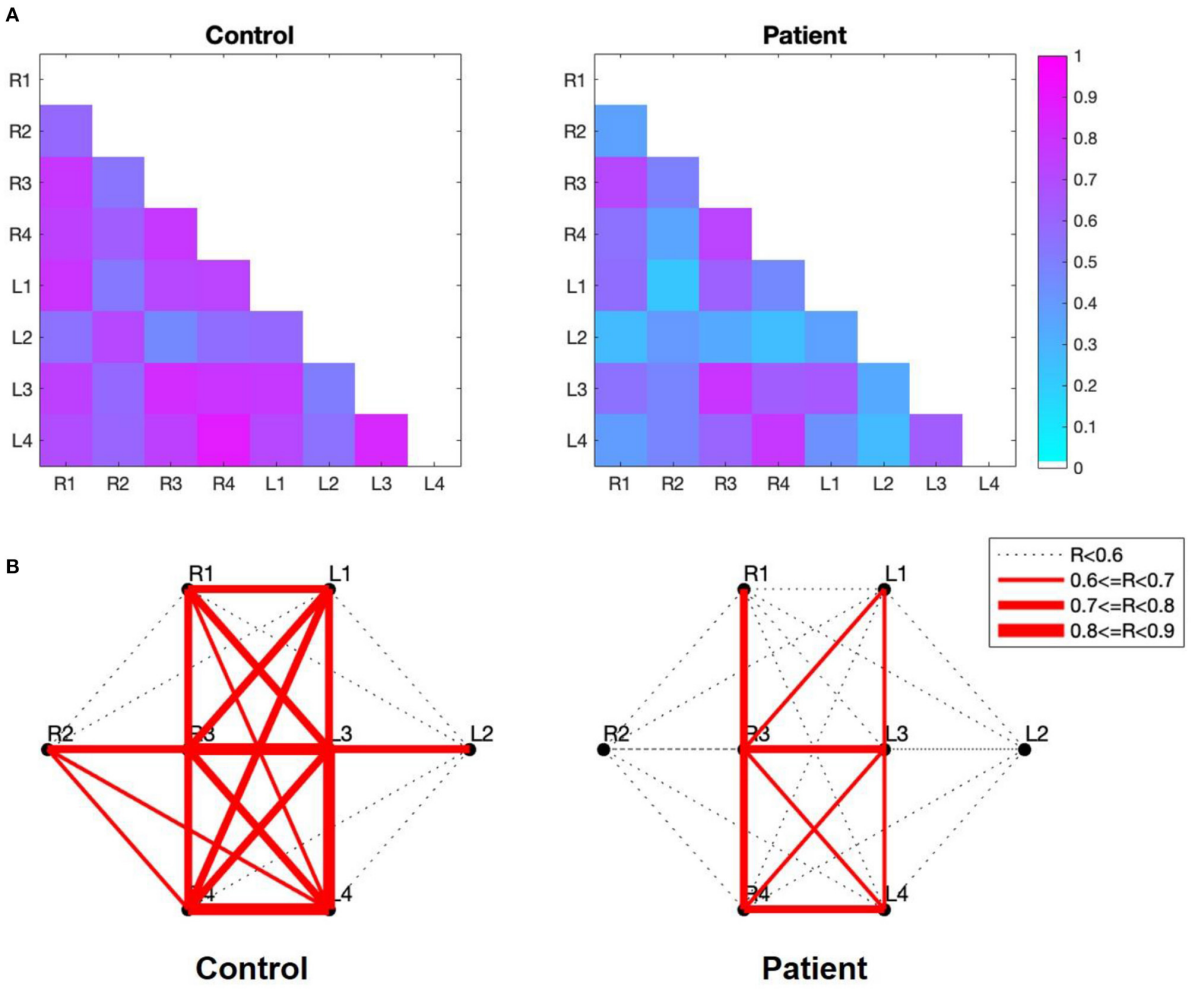
**(A)** Correlation maps of the verbal fluency task for the two groups. The image shows the lower diagonal of the cross-correlation matrix. The rows and columns of the matrix represent the subgroup, while the cells of the matrix contain the color-coded correlation coefficient of the corresponding pair. The blue cells indicate a lower between-subgroup correlation, while higher between-subgroup correlations are indicated in magenta. **(B)** Subregion-based functional connectivity The line width indicates the strength of the correlation, as determined by the correlation coefficient (R) between each pair of subregions.

The strength of the temporal correlation of hemodynamics between all pairwise combinations of the eight subgroups was calculated using Pearson's correlation coefficient. As shown in Figure 4A, the strength of the between-subgroup correlation was higher in the control group than in the patient group. The average of the correlation coefficients of the 28 pairs in the control group (0.68 ± 0.12) was higher than that of the patient group (0.49 ± 0.16). Figure 4B shows the spatial distribution of the correlations between each pair of subregions. These figures allow us to observe the areas in which connections are either stronger or weaker. Remarkably, strong interhemispheric connections (e.g., R1-L1, R2-L2, R3-L3, R4-L4) were observed in the control group, but not in the patient group.

**Figure 5:**
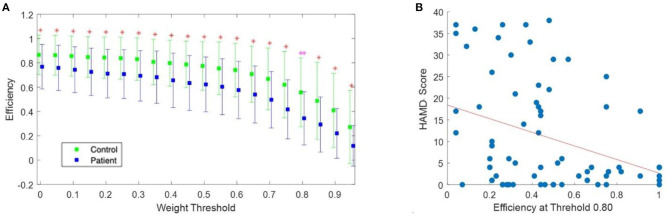
**(A)** Efficiency as a function of the threshold value. The green squares indicate efficiency in the control group while the blue squares indicate efficiency in the patient group. The error bar indicates the standard deviation. The magenta double asterisk indicates a significant difference at the level of 0.001, while red asterisks indicate a significant difference at the level of 0.05 (*p*-values were FDR-corrected). **(B)** Scatter plot of the efficiency at a threshold of 0.80 vs HAMD scores. The red line indicates the least-squares line. FDR, false discovery rate; HAMD, hamilton depression rating scale.

We tested the normality of the network measures for each threshold value and evaluated group differences using two-sample *t*-tests. First, a significant difference in the clustering coefficient was observed only at the threshold level of 0.90. The clustering coefficient at 0.9 was significantly larger in the control group (1.01 ± 0.25) than in the patient group (0.76 ± 0.43) [t (44.522) = 2.96, corrected *p* = 0.05] with a difference of 0.17 (95% CI, 0.082 to 0.43). In contrast, density and efficiency exhibited statistically significant between-group differences at all threshold levels (*p* < 0.05). The most significant difference in density was observed at a threshold level of 0.8. At this threshold level, the density in the control group (0.77 ± 0.30) was significantly higher than that in the patient group (0.55 ± 0.21) [t (72) = 3.61, corrected *p* = 0.02] with a difference of 0.22 (95% CI, 0.097 to 0.33). As shown in Figure 5A, the most significant difference in efficiency was also found at the threshold level of 0.8. At this threshold level, the efficiency in the control group (0.56 ± 0.28) was significantly higher than that in the patient group (0.34 ± 0.22) [t (71.64) = 3.66, corrected p < 0.001], with a difference of 0.058 (95% CI, 0.097 to 0.33). Moreover, we measured the correlation coefficient between the efficiency at this threshold level and the corresponding HAMD scores. A Spearman's correlation revealed a significant negative correlation between these two factors (rho = −0.30, *p* = 0.009), as shown in Figure 5B.

## Discussion

In this study, we performed functional connectivity-based comparisons between patients with MDD and healthy individuals and calculated the subregional functional connectivity using temporal correlations of the hemodynamics of every pair of subregions of the PFC using a functional connectivity approach. The functional connectivity in each group (control and patient) was visually represented by varying the thickness according to the strength of the subregional functional connectivity. Network measures were calculated to quantitatively evaluate and compare two networks, and the most informative metric was identified as the network measure representing the greatest difference between the two groups (control vs. patient).

The visualization of functional connectivity allowed us to understand subregion-wise functional connection characteristics between the two groups. We observed a clear absence of interhemispheric correlations in patients with MDD. This result is consistent with a previous study indicating that patients with MDD show interhemispheric connectivity deficits in resting-state (38).

The network measures used in our study are known to characterize basic aspects of network organization (35). Density reflects the overall wiring cost of the network. The clustering coefficient indicates network segregation, while efficiency reflects the network integration (36). Of the two measures, we focused extensively on efficiency, since it showed a significant between-group difference. We observed a strong negative correlation between fNIRS measures and the severity of depression, which is in line with other two studies (8, 9). Unlike these studies, which calculated correlations based on changes in HbO2 from a single channel (8) or an entire frontal channel (9), we used the network measure instead of HbO2 changes in this study; thus, we were able to offernew interpretations of the relationship between fNIRS measurement and depression severity. As efficiency is a measure of the effectiveness of information transfer, efficiency may play an important role in understanding decreased responsiveness in patients with MDD, including a slower reaction time (4). Although the Spearman's rho between reaction time and efficiency was not statistically significant in our study (rho = −0.21, *p* = 0.073), efficiency was the most negatively correlated network metric of the three. Future studies with a larger population may provide experimental evidence. Thus, the results so far demonstrate that these network metrics may be used as valid biomarkers of MDD.

Since most previous studies have focused primarily on resting-state functional connectivity (23–25), this study is distinct in that it suggests task-related functional connectivity. While Rosenbaum et al. have also investigated the functional connectivity during task performance as well as at rest (24, 25), the task adopted in these studies on late-life depression was a trail-making test that is often used for screening visual attention and task switching. This study uses the VFT adopted in the majority of previous studies of MDD. Therefore, we believe that our findings provide meaningful evidence that functional connectivity may be used to understand the behavioral and neurological characteristics of decreased verbal fluency in patients with MDD.

## Data Availability Statement

The datasets generated for this study cannot be made openly available due to ethical concerns. Requests to access the datasets should be directed to Seung-Hwan Lee, lshpss@hanmail.net.

## Ethics Statement

The studies involving human participants were reviewed and approved by Institutional Review Board of Inje University Ilsan Paik Hospital (2017–10–013). The patients/participants provided their written informed consent to participate in this study.

## Author's Note

This study has some limitations. Given that aging is a critical cause of cognitive decline (17) and in light of the potential need to set clinical cut-off points in rating scales for older patients (39), further investigations on age-related functional connectivity in patients with MDD are necessary. Moreover, although depression severity was not the main focus of our study, stricter criteria for enrolling patients with MDD could have provided more accurate results that better represent the characteristics of MDD.

## Author Contributions

S-YD: conceptualization, data analysis, funding acquisition, and writing – original draft. JC: protocol design, data preprocessing, and writing – review & editing. YP and SB: protocol design, investigation, and writing – review & editing. MJ and YK: data collection, writing – review & editing. S-HL: conceptualization, supervision and writing – review & editing. All authors contributed to the article and approved the submitted version.

## Conflict of Interest

JC is the inventor of continuous-wave near infrared spectroscopy technology, licensed to KAIST's spin-off company OBELAB, which focuses on non-invasive, optical brain imaging. The remaining authors declare that the research was conducted in the absence of any commercial or financial relationships that could be construed as potential conflicts of interest.
