# Supplementary material for: Prefrontal Functional Connectivity During the Verbal Fluency Task in Patients With Major Depressive Disorder: A Functional Near-Infrared Spectroscopy Study
Source: Front Psychiatry. 2021 May 21;12:659814. doi: 10.3389/fpsyt.2021.659814 (PMC8175962; doi:10.3389/fpsyt.2021.659814)
Supplement: Supplementary file 1 [file Image_1.PDF]

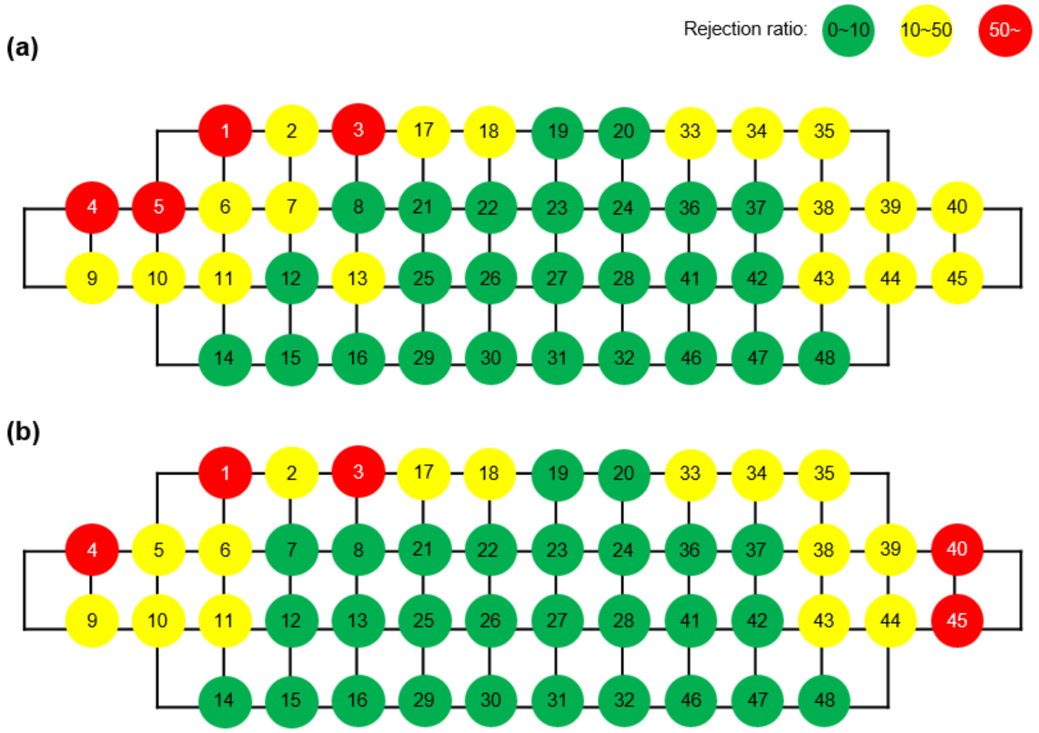

Supplementary Figure 1. Location of NIRS channels with color-scaled rejection ratios of (a) Control and (b) Patient groups, respectively. In a numbered NIRS channel, green, yellow, and red colors indicate 0-10. 10-50, and higher than 50% rejection ratios.
