# Supplementary material for: Prefrontal Functional Connectivity During the Verbal Fluency Task in Patients With Major Depressive Disorder: A Functional Near-Infrared Spectroscopy Study
Source: Front Psychiatry. 2021 May 21;12:659814. doi: 10.3389/fpsyt.2021.659814 (PMC8175962; doi:10.3389/fpsyt.2021.659814)
Supplement: Supplementary file 2 [file Image_2.PDF]

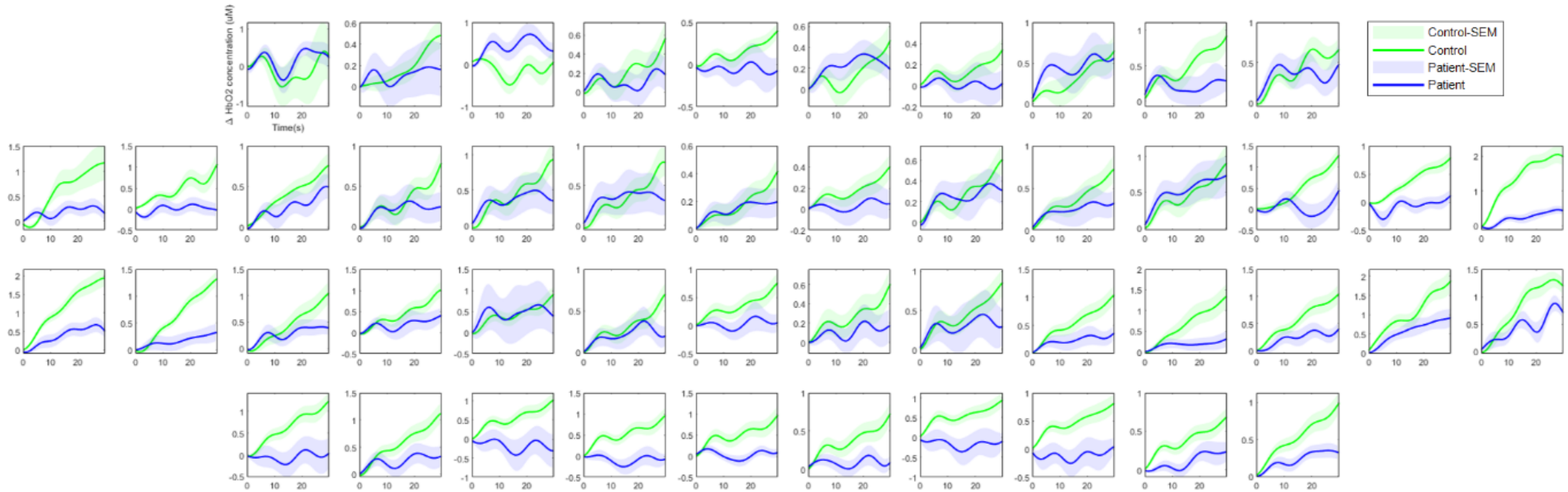

Supplementary Figure 2. The traces of the oxygenated hemoglobin concentration (uM) for 48 channels with standard errors of the mean as shaded region.
